# Supplementary material for: “I sometimes feel like I can’t win!”: An exploratory mixed-methods study of women’s body image and experiences of exercising in gym settings
Source: PLoS One. 2025 Jan 29;20(1):e0316756. doi: 10.1371/journal.pone.0316756 (PMC11778772; doi:10.1371/journal.pone.0316756)
Supplement: S1 Appendix — (DOCX) [file pone.0316756.s001.docx]

**Supplementary Materials**

**Appendix A – Study Survey**

**SCREENING**

I identify as a woman.

- Yes (1)
- No (2)

I am 18 years old or above.

- Yes (1)
- No (2)

Which best describes you?

- I currently go to the gym (1)
- I used to go to the gym (2)
- I've never gone to the gym (3)

**EXERCISE HABITS**

How often do you go to the gym on average?

- Once per week (1)
- 2–3 times per week (2)
- 4–6 times per week (3)
- Every day (4)
- Prefer not to say (5)

How much physical activity do you do on average?

- Less than 1 hour per week (1)
- 1–3 hours per week (2)
- 3–6 hours per week (3)
- 7–9 hours per week (4)
- 10+ hours per week (5)
- Prefer not to say (6)

What is the predominant type of exercise you do each week?

- Cardio (e.g., running, swimming, cycling) (1)
- Strength training (e.g., weight lifting) (2)
- Flexibility training (e.g., yoga) (3)
- Other, please specify: (4) __________________________________________________
- Prefer not to say (5)

**PHYSICAL APPEARANCE**

Do you have any of the below skin concerns?

- Facial acne (1)
- Spots on shoulders/back/chest (2)
- Stretch marks (3)
- Eczema (4)
- Other, please specify: (5) __________________________________________________
- None (6)
- Prefer not to say (7)

Does this concern impact how you feel about exercising in a gym?

- Yes (1)
- No (2)
- Unsure (3)
- Prefer not to say (4)

How much do you agree with the following statement?
*“I will only wear shorts to the gym if I have fake tanned my legs.”*

|  | 1 (1) | 2 (2) | 3 (3) | 4 (4) | 5 (5) |  |
| --- | --- | --- | --- | --- | --- | --- |
| Strongly disagree |  |  |  |  |  | Strongly agree |

Do you typically wear makeup to the gym?

- Yes, I wear my normal make up to the gym (1)
- Yes, but I only use light makeup like concealer (2)
- No, but I used to wear makeup to the gym (3)
- No, I never put on makeup to go to the gym (4)
- Prefer not to say (5)

How confident do you feel about going to the gym without wearing make-up?

|  | 1 (1) | 2 (2) | 3 (3) | 4 (4) | 5 (5) |  |
| --- | --- | --- | --- | --- | --- | --- |
| Not confident at all |  |  |  |  |  | Very confident |

Do you typically style your hair before going to the gym?

- Yes, I use a styler on my hair before the gym (e.g., curling iron) (1)
- Yes, but only some styling product (e.g., hair spray) (2)
- No, but I used to style my hair before going to the gym (3)
- No, I never style my hair before going to the gym (4)
- Prefer not to say (5)

How comfortable do you feel lifting your arms overhead in the gym if you have not shaved your under arms?

|  | 1 (1) | 2 (2) | 3 (3) | 4 (4) | 5 (5) |  |
| --- | --- | --- | --- | --- | --- | --- |
| Not comfortable at all |  |  |  |  |  | Makes no difference to me |

What part of your body do you feel **most** confident about?

- Arms (1)
- Back (2)
- Chest (3)
- Face (4)
- Legs (5)
- Shoulders (6)
- Stomach (7)
- Waist (8)
- Bum (9)
- Other, please specify: (10) __________________________________________________
- None (11)
- Prefer not to say (12)

What part of your body do you feel **least** confident about?

- Arms (1)
- Back (2)
- Chest (3)
- Face (4)
- Legs (5)
- Shoulders (6)
- Stomach (7)
- Waist (8)
- Bum (9)
- Other, please specify: (10) __________________________________________________
- None (11)
- Prefer not to say (12)

Are there certain body parts where you do **want** to build muscle?

- Arms (1)
- Back (2)
- Chest (3)
- Face (4)
- Legs (5)
- Shoulders (6)
- Stomach (7)
- Waist (8)
- Bum (9)
- Other, please specify: (10) __________________________________________________
- None (11)
- Prefer not to say (12)

Are there certain exercises you **do** so that you can build muscle on these body parts?

________________________________________________________________

Are there certain body parts where you **do not** want to build muscle?

- Arms (1)
- Back (2)
- Chest (3)
- Face (4)
- Legs (5)
- Shoulders (6)
- Stomach (7)
- Waist (8)
- Bum (9)
- Other, please specify: (10) __________________________________________________
- None (11)
- Prefer not to say (12)

Are there certain exercises you **avoid** so that you don’t build muscle on these body parts?

________________________________________________________________

Are you ever concerned you will become “too” muscular through exercising?

- Yes (1)
- No (2)
- Unsure (3)
- Prefer not to say (4)

Why are you concerned about becoming “too” muscular? *Please select all that apply.*

- I worry I will not look “feminine” enough (1)
- I don't think muscles on women are attractive (2)
- I worry about what other people will think of me (3)
- Other, please specify: (4) __________________________________________________
- Prefer not to say (5)

If there is something we have not asked about, please tell us what else do you think or worry about related to your **physical appearance** while exercising in the gym.

________________________________________________________________

**GYM ATTIRE**

Do you wear activewear (clothing you wear to the gym; e.g., leggings) outside the gym when not exercising?

- No, I only wear activewear in the gym (1)
- Yes, I wear activewear in and out of the gym (2)
- Yes, I don’t go to the gym and still wear activewear in my daily life (3)
- Prefer not to say (4)

How comfortable do you feel wearing shorts to the gym?

|  | 1 (1) | 2 (2) | 3 (3) | 4 (4) | 5 (5) |  |
| --- | --- | --- | --- | --- | --- | --- |
| Not comfortable at all |  |  |  |  |  | Extremely comfortable |

Can you please briefly expand on your answer? *E.g., why you do/don’t feel comfortable wearing shorts to the gym.*

________________________________________________________________

If you had to choose between wearing a pair of **white** shorts or a pair of **black** shorts to the gym, which would you choose?

- White (1)
- Black (2)
- Either (4)
- Prefer not to say (3)

Why would you choose **not** to wear white shorts to the gym? *Please select all that apply.*

- I would worry about period stains (1)
- I would worry about the shorts being see-through (2)
- I would worry about showing my cellulite (3)
- Black is more “flattering” (4)
- Other, please specify: (5) __________________________________________________
- Prefer not to say (6)

How comfortable do you feel wearing crop tops (or similar clothing that shows the stomach) to the gym?

|  | 1 (1) | 2 (2) | 3 (3) | 4 (4) | 5 (5) |  |
| --- | --- | --- | --- | --- | --- | --- |
| Not comfortable at all |  |  |  |  |  | Extremely comfortable |

Can you please briefly expand on your answer? *E.g., why you do/don’t feel comfortable wearing a crop top to the gym.*

________________________________________________________________

Which type of clothing **do you more commonly wear** to the gym?

- Tight, form-fitting clothing (1)
- Oversized, concealing clothing (2)
- Prefer not to say (3)

Which type of clothing **would you like to wear** to the gym?

- Tight, form-fitting clothing (1)
- Oversized, concealing clothing (2)
- Prefer not to say (3)

When deciding what to wear to the gym, which is typically the priority?

- Clothing that makes me comfortable (1)
- Clothing that makes me confident (2)
- Clothing that makes me look good (3)
- Prefer not to say (4)

Do you think you perform better when you wear **clothing you feel confident in**?

- Yes (1)
- No (2)
- Unsure (3)
- Prefer not to say (4)

Do you think you perform better in more **form-fitting clothing**?

- Yes (1)
- No (2)
- Unsure (3)
- Prefer not to say (4)

If you menstruate, do you change **what you wear** to the gym when you have your period?

- Yes (1)
- No (2)
- Unsure (3)
- Does not apply to me (4)
- Prefer not to say (5)

What do you wear to the gym when you have your period? *Please select all that apply.*

- Looser clothing to relieve pressure if cramping (1)
- Less exposing clothing due to fear of leaking (2)
- Darker clothing due to fear of leaking (3)
- Looser clothing due to feeling bloated (4)
- More supportive bras if breasts are sensitive (5)
- Other, please specify: (6) __________________________________________________
- Prefer not to say (7)

If you menstruate, do you change **what exercises you do** in the gym when you have your period?

- Yes (1)
- No (2)
- Unsure (3)
- Does not apply to me (4)
- Prefer not to say (5)

How concerned are you with sweating and having sweat marks in the gym?

|  | 1 (1) | 2 (2) | 3 (3) | 4 (4) | 5 (5) |  |
| --- | --- | --- | --- | --- | --- | --- |
| Not concerned at all |  |  |  |  |  | Extremely concerned |

How likely are you to wear specific clothing materials and/or colours to minimise sweat marks when exercising in the gym?

|  | 1 (1) | 2 (2) | 3 (3) | 4 (4) | 5 (5) |  |
| --- | --- | --- | --- | --- | --- | --- |
| Not likely at all |  |  |  |  |  | Extremely likely |

How do you typically shop for activewear?

- Online (1)
- In store (2)
- Prefer not to say (3)

How does shopping for activewear online make you feel about your body?

|  | 1 (1) | 2 (2) | 3 (3) | 4 (4) | 5 (5) |  |
| --- | --- | --- | --- | --- | --- | --- |
| Really bad about my body |  |  |  |  |  | Really good about my body |

Do you think there is sufficient choice in activewear for your body size and shape?

- Yes (1)
- No (2)
- Unsure (3)
- Prefer not to say (4)

Are there any particular items that are challenging to purchase? *E.g., leggings, tops, sports bras, etc.*

________________________________________________________________

Do you wear leggings to the gym?

- Yes (1)
- No (2)
- Prefer not to say (3)

Please tick **up to 3 features** that are most important when buying gym leggings.

- High waisted (1)
- Compression (2)
- Ruching to emphasise glutes (3)
- Seamless (4)
- Squat proof (5)
- Contouring (6)
- Sweat wicking (7)
- Pockets (8)
- Other, please specify: (9) __________________________________________________
- Prefer not to say (10)

As leggings are naturally form-fitting, are you concerned with showing underwear lines/seams when exercising in the gym? *E.g., wearing seamless underwear, etc.*

- Yes (1)
- No (2)
- Unsure (3)
- Prefer not to say (4)

What is the main reason you are concerned with showing underwear lines/seams?

- Brought up told that women shouldn’t have VPLs (visible panty lines) (1)
- Seamless are more comfortable (2)
- Leggings look better without underwear seams (3)
- Other, please specify: (4) __________________________________________________
- Prefer not to say (5)

When buying tops to wear to the gym, please select if you prefer the below body parts to be on show or covered.

|  | On show (1) | Covered (2) |
| --- | --- | --- |
| Stomach (1) |  |  |
| Shoulders (2) |  |  |
| Arms (3) |  |  |
| Back (4) |  |  |
| Chest/cleavage (5) |  |  |

If you had to choose between wearing a **light coloured** sports bra (e.g., white) or a **dark coloured** sports bra (e.g., black) to the gym, which would you choose?

- Light coloured (1)
- Dark coloured (2)
- Either (4)
- Prefer not to say (3)

Why would you choose **not** to wear a light coloured sports bra to the gym? *Please select all that apply.*

- Dark colours are more “flattering” (1)
- I would worry about showing sweat marks (2)
- I would worry about my nipples showing (3)
- Other, please specify: (4) __________________________________________________
- Prefer not to say (5)

Do you ever choose to workout in a sports bra only in the gym?

- Yes (1)
- No (2)
- Prefer not to say (3)

Please select which features are most important when buying sports bras.

|  | 1 (1) | 2 (2) |  |
| --- | --- | --- | --- |
| Less supportive |  |  | More supportive |
| Less coverage |  |  | More coverage |
| No padding |  |  | Padding |
| Covered back |  |  | Low or open back |
| High neck |  |  | V-neck or plunge neck |

If you see a woman in the gym wearing clothing that is exposing or form-fitting, how does it make you feel?

- Liberated (1)
- Don’t care (2)
- Threatened (3)
- Jealous (4)
- Other, please specify: (5) __________________________________________________
- Prefer not to say (6)

Do you consider potential dangers when choosing what to wear to the gym? *E.g., unwanted attention, comments or physical touch.*

- Yes (1)
- No (2)
- Unsure (3)
- Prefer not to say (4)

How much do you agree with this statement?

*“Sometimes I focus more on how I look in the gym than on the exercise I am performing.”*

|  | 1 (1) | 2 (2) | 3 (3) | 4 (4) | 5 (5) |  |
| --- | --- | --- | --- | --- | --- | --- |
| Strongly disagree |  |  |  |  |  | Strongly agree |

If there is anything we haven’t covered, what else do you think or worry about related to your **gym clothing** while exercising in the gym?

________________________________________________________________

**GYM SPACE**

What is your preferred type of gym?

- Commercial chain gym (e.g., Ben Dunne, JD Gyms, Gold’s Gym) (1)
- Personal training/Small group training gym (2)
- Women’s only gym (3)
- HIIT class gym (e.g., F45, Barry’s Camp) (4)
- Yoga/Pilates studio (5)
- CrossFit box (6)
- Sport specific gym (e.g., boxing, weight lifting) (7)
- Other, please specify: (8) __________________________________________________
- Prefer not to say (9)

Which type of gym do you prefer?

- Open-plan gym spaces (e.g., a large weights and/or cardio hall) (1)
- Closed off spaces (e.g., studio or matted area of the gym) (2)
- Prefer not to say (3)

When you go to the gym, do you typically:

- Go alone (1)
- Go with a friend/family member (2)
- Take part in a class (3)
- Exercise with a trainer (4)
- Other, please specify: (5) __________________________________________________
- Prefer not to say (6)

How do you feel about exercising in front of mirrors?

- I hate exercising in front of mirrors (1)
- I’d prefer to not exercise in front of a mirror (2)
- I don’t care either way (3)
- I’d prefer to exercise in front of a mirror (4)
- I love exercising in front of a mirror (5)
- Prefer not to say (6)

Can you please expand on your answer? *E.g., why you do/don’t like exercising in front of mirrors.*

________________________________________________________________

What time do you typically go to the gym?

- Before 7am (1)
- 7am–9am (2)
- 9am–12pm (3)
- 12pm–3pm (4)
- 3pm–6pm (5)
- 6pm–8pm (6)
- After 8pm (7)
- Prefer not to say (8)

How busy is the gym at this time?

- It’s very quiet (1)
- It’s quiet (2)
- It’s moderately busy (3)
- It’s busy (4)
- It’s very busy (5)
- Prefer not to say (6)

Why do you go to the gym at this time?

- It fits best with my other commitments (e.g., work) (1)
- It is the quietest time (2)
- That is when the classes I go to are scheduled (3)
- That is when my friends train (4)
- Other, please specify: (5) __________________________________________________
- Prefer not to say (6)

What areas of the gym do you feel **most** comfortable using?

- Cardio equipment (1)
- Free weight area (e.g., dumbbells, squat rack) (2)
- Resistance machines (e.g., cables, machine row, machine leg press) (3)
- Studio/astro space (e.g., medicine balls, kettlebells, ropes, prowler) (4)
- Other, please specify: (5) __________________________________________________
- Prefer not to say (6)

Can you please expand on why you feel most comfortable here? *Please select all that apply.*

- More quiet (1)
- Less intimidating (2)
- Prefer the exercise (3)
- I know how to use the equipment (4)
- Other, please specify: (5) __________________________________________________
- Prefer not to say (6)

What areas of the gym do you feel **least** comfortable using?

- Cardio equipment (1)
- Free weight area (e.g., dumbbells, squat rack) (2)
- Resistance machines (e.g., cables, machine row, machine leg press) (3)
- Studio/astro space (e.g., medicine balls, kettlebells, ropes, prowler) (4)
- Other, please specify: (5) __________________________________________________
- Prefer not to say (6)

Can you please expand on why you feel least comfortable here? *Please select all that apply.*

- More busy (1)
- More intimidating (2)
- I don’t enjoy the type of exercise (3)
- I don’t know how to use the equipment (4)
- Other, please specify: (5) __________________________________________________
- Prefer not to say (6)

What gym equipment do you feel **most** comfortable using? *Please select all that apply.*

- Free weights (e.g., dumbbells, barbells) (1)
- Resistance machines (2)
- Conditioning equipment (e.g., medicine ball, plyometric boxes, prowler) (3)
- Cardio equipment (e.g., treadmill, bicycle) (4)
- No equipment (e.g., body weight exercise, yoga mat) (5)
- Other, please specify: (6) __________________________________________________
- Prefer not to say (7)

Can you please expand on why you feel most comfortable using this equipment?

________________________________________________________________

What gym equipment do you feel **least** comfortable using? *Please select all that apply.*

- Free weights (e.g., dumbbells, barbells) (1)
- Resistance machines (2)
- Conditioning equipment (e.g., medicine ball, plyometric boxes, prowler) (3)
- Cardio equipment (e.g., treadmill, bicycle) (4)
- No equipment (e.g., body weight exercise, yoga mat) (5)
- Other, please specify: (6) __________________________________________________
- Prefer not to say (7)

Can you please expand on why you feel least comfortable using this equipment?

________________________________________________________________

Are there any particular exercises that make you feel **most** confident about your body? *Please select all that apply.*

- Cardio based exercise (e.g., running, cycling, swimming) (1)
- Flexibility focused exercise (e.g., yoga) (2)
- Hybrid style exercise (e.g., Crossfit, F45, circuit-style training) (3)
- Sport specific exercise (e.g., boxing, dancing) (4)
- Strength based exercise (e.g., weight lifting) (5)
- None (6)
- Other, please specify: (7) __________________________________________________
- Prefer not to say (8)

Are there any particular exercises that make you feel **least** confident about your body? *Please select all that apply.*

- Cardio based exercise (e.g., running, cycling, swimming) (1)
- Flexibility focused exercise (e.g., yoga) (2)
- Hybrid style exercise (e.g., Crossfit, F45, circuit-style training) (3)
- Sport specific exercise (e.g., boxing, dancing) (4)
- Strength based exercise (e.g., weight lifting) (5)
- None (6)
- Other, please specify: (7) __________________________________________________
- Prefer not to say (8)

If there is something we have not asked, what else do you think or worry about related to the **gym environment**?

________________________________________________________________

**INTERACTIONS WITH OTHERS IN THE GYM**

Who do you feel more **intimidated** by when exercising in the gym?

- I feel more intimidated by men (1)
- I feel more intimidated by women (2)
- I feel equally intimidated by both men and women (3)
- I never feel intimidated (4)
- Prefer not to say (5)

Who do you feel more **judged** by when exercising in the gym?

- I feel more judged by men (1)
- I feel more judged by women (2)
- I feel equally judged by both men and women (3)
- I never feel judged (4)
- Prefer not to say (5)

If a **woman** was staring at you in the gym, what words would you use to describe how it would make you feel?

- Empowered (1)
- Friendly (2)
- Judged (3)
- Intimidated (4)
- Curious (5)
- Angry (6)
- Other, please specify: (7) __________________________________________________
- Prefer not to say (8)

If a **man** was staring at you in the gym, what words would you use to describe how it would make you feel?

- Empowered (1)
- Friendly (2)
- Judged (3)
- Intimidated (4)
- Curious (5)
- Angry (6)
- Other, please specify: (7) __________________________________________________
- Prefer not to say (8)

***This next set of questions is related to harassment in the gym space, including verbal comments, physical and/or sexual harassment. If you do not want to answer these questions, please click “Skip” below.***

- Continue to questions (1)
- Skip this section (2)

Have you ever received an unsolicited comment **complimenting** your appearance while in the gym?

- Yes (1)
- No (2)
- Unsure (3)
- Prefer not to say (4)

Who made the comment?

- A gym staff member (man) (1)
- A gym staff member (woman) (2)
- Another person exercising (man) (3)
- Another person exercising (woman) (4)
- Other, please specify: (5) __________________________________________________
- Prefer not to say (6)

How did the comment make you feel?

________________________________________________________________

If you feel comfortable sharing, what was the comment?

________________________________________________________________

Have you ever received an unsolicited comment **insulting** your appearance while in the gym?

- Yes (1)
- No (2)
- Unsure (3)
- Prefer not to say (4)

Who made the comment?

- A gym staff member (man) (1)
- A gym staff member (woman) (2)
- Another person exercising (man) (3)
- Another person exercising (woman) (4)
- Other, please specify: (5) __________________________________________________
- Prefer not to say (6)

How did the comment make you feel?

________________________________________________________________

If you feel comfortable sharing, what was the comment?

________________________________________________________________

Have you experienced any form of physical or sexual harassment while in the gym? *Physical harassment involves unwanted, intentional contact or threatening gestures.*

- Yes, I have been personally harassed (1)
- Yes, I have witnessed someone else being harassed (2)
- No, I have not been harassed nor witnessed it (3)
- Unsure (4)
- Prefer not to say (5)

Who was the person doing the harassing?

- A gym staff member (man) (1)
- A gym staff member (woman) (2)
- Another person exercising (man) (3)
- Another person exercising (woman) (4)
- Other, please specify: (5) __________________________________________________
- Prefer not to say (6)

Did you report the event?

- Yes (1)
- No (2)
- Prefer not to say (3)

Did you change your gym behaviour after this harassment?

- Yes, I stopped exercising altogether (1)
- Yes, I changed the gym I go to (2)
- Yes, I changed my gym routine and/or schedule (3)
- Yes, I avoid certain areas of the gym (4)
- Yes, I changed my clothing and/or appearance in the gym (5)
- No, I did not change my behaviour (6)
- Prefer not to say (7)

If there is something we have not asked, what other thoughts do you have about how **interacting with other people** makes you feel in the gym environment?

________________________________________________________________

**DEMOGRAPHICS**

What is your age?

- Under 25 years (1)
- 25–29 years (2)
- 30–39 years (3)
- 40–49 years (4)
- 50–59 years (5)
- 60 years and above (6)
- Prefer not to say (7)

What country are you currently residing in?

________________________________________________________________

What country were you born in?

________________________________________________________________

What ethnicity best describes you?

- Asian or Asian British/Irish (1)
- Black, Black British/Irish, Caribbean or African (2)
- Mixed or multiple ethnic groups (3)
- White/Caucasian (4)
- Other, please specify: (5) __________________________________________________
- Prefer not to say (6)

Do you consider yourself to have a disability?

- Yes, I have a disability (1)
- No, I do not have a disability (2)
- Prefer not to say (3)

What is your sexual orientation?

- Asexual (1)
- Bisexual (2)
- Lesbian (3)
- Queer (4)
- Straight (heterosexual) (5)
- Other, please specify: (6) __________________________________________________
- Prefer not to say (7)

How do you perceive yourself?

- “Underweight” (1)
- “Normal weight” (2)
- “Overweight” (3)
- Prefer not to say (4)

Are you currently trying to change your weight?

- Yes, I am trying to lose weight (1)
- Yes, I am trying to gain weight (2)
- No, I’m not trying to lose or gain weight (3)
- Prefer not to say (4)
